# Main-Group Metal Atoms Anchored on Borophene/Al(111) as an Efficient Catalyst for Electrocatalytic Nitrate Pollutant Reduction Reactions: A First-Principle Perspective

**DOI:** 10.3390/molecules31213983

**Published:** 2026-11-04

**Authors:** Zhenhua Liu, Tian Gao, He Wang, Yueyue Zhang, Yutong Han, Yixuan Gu, Honghui Teng, Hao Sun

**Affiliations:** 1 College of Engineering, Jilin Normal University, Siping 136000, China; liuzhenhua@jlnu.edu.cn; 2 College of Chemistry, Jilin Normal University, Siping 136000, China; 3 Jilin Provincial Key Laboratory of Emerging Contaminants Identification and Control, Jilin Normal University, Siping 136000, China; 4 Institute of Functional Material Chemistry, Faculty of Chemistry, National & Local United Engineering Lab for Power Battery, Northeast Normal University, Changchun 130024, China; gaotian199906@163.com (T.G.); zhangyueyue@nenu.edu.cn (Y.Z.); hanyt613@nenu.edu.cn (Y.H.); guyx224@nenu.edu.cn (Y.G.); 5 Key Laboratory of Preparation and Application of Environmental Friendly Materials, Ministry of Education, Jilin Normal University, Changchun 130103, China; wanghe@jlnu.edu.cn (H.W.)

**Keywords:** electrochemical nitrate reduction reaction, single-atom catalyst, main-group metal catalyst, honeycomb borophene

## Abstract

Ammonia is essential for sustainable development, and electrochemical nitrate reduction (e-NitRR) enables simultaneous nitrate remediation and ammonia recovery. Here, main-group single atoms anchored on honeycomb borophene/Al(111) (M-HB/Al(111)) were systematically investigated. Screening based on binding energies, aggregation tendencies, and molecular dynamics identified nine stable configurations among eleven candidates. Li-HB/Al(111) and Na-HB/Al(111) exhibited outstanding activity, with limiting potentials of −0.04 and −0.13 V, respectively. Charge density and projected density-of-states analyses revealed an ‘‘ionic-bond fixation and electron bridge’’ mechanism, in which metal sites stabilize NO3- through ionic interactions and facilitate electron transfer from the substrate to intermediates. Moreover, a descriptor integrating NO_3_^−^ adsorption free energy, atomic radius, and valence electron number correlated strongly with catalytic activity. These findings identify efficient main-group electrocatalysts, clarify the atomic-scale reaction mechanism, and establish quantitative principles for rational e-NitRR catalyst design, providing theoretical guidance for nitrate valorization and sustainable ammonia production in practical aqueous treatment systems.

## 1. Introduction

Reducing nitrogen-containing pollutants, specifically nitrate, to ammonia and subsequently using the ammonia for energy production represents a significant approach that combines both environmental and energy benefits. Ammonia (NH_3_) is a significant carbon-neutral energy carrier, contributing immeasurably to sustaining population growth and ecological equilibrium [1,2,3]. The predominant approach to producing NH_3_ is the Haber–Bosch process (HBP). However, this method is characterized by severe reaction conditions and substantial energy consumption, which are incongruent with an environmentally friendly and sustainable strategy [4]. Hence, it is crucial to explore novel synthetic schemes for NH_3_.

Various catalytic processes for NH_3_ synthesis, such as photocatalysis, electrocatalysis, and enzyme catalysis, have been researched and developed in recent years [5,6,7]. Among these, the electrochemical synthesis of NH_3_ using existing resources in nature and renewable energy shows great potential as a viable alternative approach to address existing challenges in the NH_3_ industry [8]. Generally, electrochemical processes for synthesizing NH_3_ can be categorized into three types: (1) the electrocatalytic nitrogen reduction reaction (e-NRR), (2) the lithium-mediated nitrogen reduction reaction (Li-NRR), and (3) electrocatalytic reduction reactions of nitrate (NO_3_^−^) or nitrite (NO_2_^−^) (e-NitRRs) [9,10,11]. Regardless, the inherent inertness of N≡N (bond energy: 934 KJ/mol) and the high ionization energy of N_2_ molecules (15.58 eV) are obstacles that must be overcome in electrochemical nitrogen reduction reactions (e-NRRs) in order to obtain a higher Faradaic Efficiency (FE) of the reaction [12,13]. It is worth noting that the higher solubility of nitrate in water and the lower N=O bond can make e-NitRRs more likely to occur. Also, reducing nitrate levels can help address the large amount of nitrite in wastewater, turning waste into a valuable resource [14,15]. Hence, e-NitRRs have received increasing attention in recent years [9,10,11]. Currently, most electrochemical reactions involving water as a solvent are influenced by the HER (hydrogen evolution reaction) as a competing reaction, leading to decreased reaction selectivity [12,13]. Therefore, the selection of catalysts is crucial for e-NitRR application.

Currently, single-atom catalysts (SACs) have attracted significant interest in the field of catalysis due to their exceptional atomic utilization efficiency and catalytic ability [16,17]. Among these, transition metal (TM) SACs based on two-dimensional materials are particularly appealing. For instance, Qiao et al., by constructing a transition metal single-atom catalyst (TM-SAC) system loaded with nitrogen-doped carbon materials, revealed that the role of metal centers is to affect the population of bonding/antibonding orbitals and regulate the adsorption energy ratio of intermediates [18]. However, current TM catalysts still have some drawbacks. On the one hand, TM catalysts release metal ions during the reaction process, which in turn pollutes the environment. On the other hand, the d orbital electrons of TM atoms are easily bound to H atoms, triggering competition with the hydrogen evolution reaction (HER) [19,20]. Therefore, TM-SACs are not long-term strategies for e-NitRRs from the perspectives of cost, price, and environmental pollution, and it is more practical to develop main-group metal SACs.

Borophene, as the lightest and thinnest material, is a rising star in the two-dimensional material family, possessing properties such as high conductivity, thermal stability, and anisotropy [21,22,23]. Since the successful synthesis of striped-phase borophene on Ag(111) substrate in 2015 [24], a series of experimental syntheses of borophene such as β [25], x [26], and honeycomb [27] phases have been reported. Honeycomb borophene (HB) belongs to a relatively special type within the borophene groups. The unique hexagonal honeycomb structure of HB naturally endows it with a Dirac-cone band structure [28]. Therefore, most of the novel quantum effects exhibited in graphene are expected to be reflected in HB [27,29]. Since the outermost layer of the B atom has one electron less than the C atom, HB has an electron-deficient structure and cannot reach a stable state in terms of energy. Therefore, HB requires a substrate to supply electrons for stabilization [29]. The first synthesis of HB was conducted in 2018. Wu et al. utilized a single-crystal Al(111) substrate and successfully synthesized HB thin films by precisely controlling the growth process [27].

In this work, we constructed SACs named M-HB/Al(111), composed of a series of main-group metal atoms (M=Li, Na, K, Rb, Be, Mg, Ca, Sr, Al, Ga, Ge) adsorbed on the surface of an HB/Al (111) heterostructure, through first-principles calculations. A comprehensive evaluation was conducted on the stability, conductivity and catalytic activity of these candidate catalysts for e-NitRRs. Finally, Li-HB/Al(111) and Na-HB/Al(111) catalysts were confirmed to have superior catalytic activity over others in e-NitRRs. They exhibited reaction limiting potentials (U_L_) of −0.04 V and −0.13 V, respectively. At the same time, their HER requires a larger reaction free-energy change compared to e-NitRRs. The positive hydrogen adsorption free energies suggest a thermodynamic tendency to suppress the competing HER under the adopted reference conditions.

## 2. Theoretical Basis

The density functional theory (DFT) computations conducted in this study were carried out using the Vienna Ab Initio simulation package (VASP) [28]. Exchange-related interactions were modeled using the Generalized Gradient Approximation (GGA) method, which originates from the Perdew Burke Ernzerhof (PBE) functional [29], while electronic interactions were described using Projection Enhanced Waves (PAWs) [30]. The cutoff energy of the plane wave was set to 500 eV (Li was set to 600 eV). All structures were subjected to a force threshold of −0.01 eV on each atom. In addition, energy convergence criteria were established at 10^−5^ eV. To characterize the van der Waals (vdW) interaction, the DFT-D3 method developed by Grimme was used for calculations [31]. A vertical vacuum space of 15 Å was implemented to minimize the interaction between adjacent repeating slabs. For structure relaxation and density of states, we utilized Monkhorst–Pack k-point grids with meshes of 3 × 3 × 1 and 3 × 3 × 4, respectively, to sample the Brillouin zone [32]. The Bader charge population was employed to examine the distribution and transfer of atomic charges [33], while VASPKIT (version 1.5.0) software was utilized to calculate the values of zero-point energy and entropy [34].

The binding energy (E_b_) was calculated using the following formula to assess the stability of M-HB/Al (111):

E_b_ = E_M-HB/Al(111)_ − E_M_ − E_HB/Al(111)_ (1)

Here, E_M-HB/Al(111)_, E_M_, and E_HB/Al(111)_ represent the electronic energy of the M-HB/Al(111) catalyst, a single metal atom, and the HB/Al(111) heterostructure, respectively.

The Computational Hydrogen Electrode (CHE) model, established by Nørskov et al. [35,36,37], calculated the free-energy change (ΔG) for each step of the e-NitRR:

ΔG = ΔE + ΔE_ZPE_ − TΔS + ΔG_U_ + ΔG_pH_ (2)

ΔE represents the electron energy calculated by DFT. ΔE_ZPE_, T, and ΔS represent the changes in zero-point energy, temperature at 298.15 K, and entropy, respectively. ΔG_U_ represents the impact of the applied potential U on the system, which can be expressed as ΔG_U_ = −eU, where e is the number of transferred electrons. Therefore, there is a correlation between U_L_ and ΔG_max_, where ΔG_max_ is the Gibbs free-energy change of the potential-determining step (PDS), which can be expressed as UL = −ΔG_max_/e. At the limit of its potential, the entire reaction pathway displays exothermic behavior. The initial screening was performed at pH = 0. Additional CHE-based free-energy profiles were constructed at pH = 7 for Li-HB/Al(111). At 298.15 K and a fixed potential of U = 0 V versus SHE, the pH correction was approximately 0.414 eV per proton consumed. Each state was corrected according to the cumulative number of protons consumed relative to the initial state. The original reaction sequence and reference-state conventions were retained. For equal proton–electron transfer steps, the explicit pH contribution cancels upon comparison at the same RHE potential within the ideal CHE approximation.

Explicit water calculations were performed for Li-HB/Al(111) using an interfacial model containing 24 H_2_O molecules. Reaction free energies were evaluated using consistent stoichiometry, reference states, and free-energy corrections for the water-containing surface and intermediates.

## 3. Results and Discussion

### 3.1. Structure and Stability of M-HB/Al(111)

**Figure 1:**
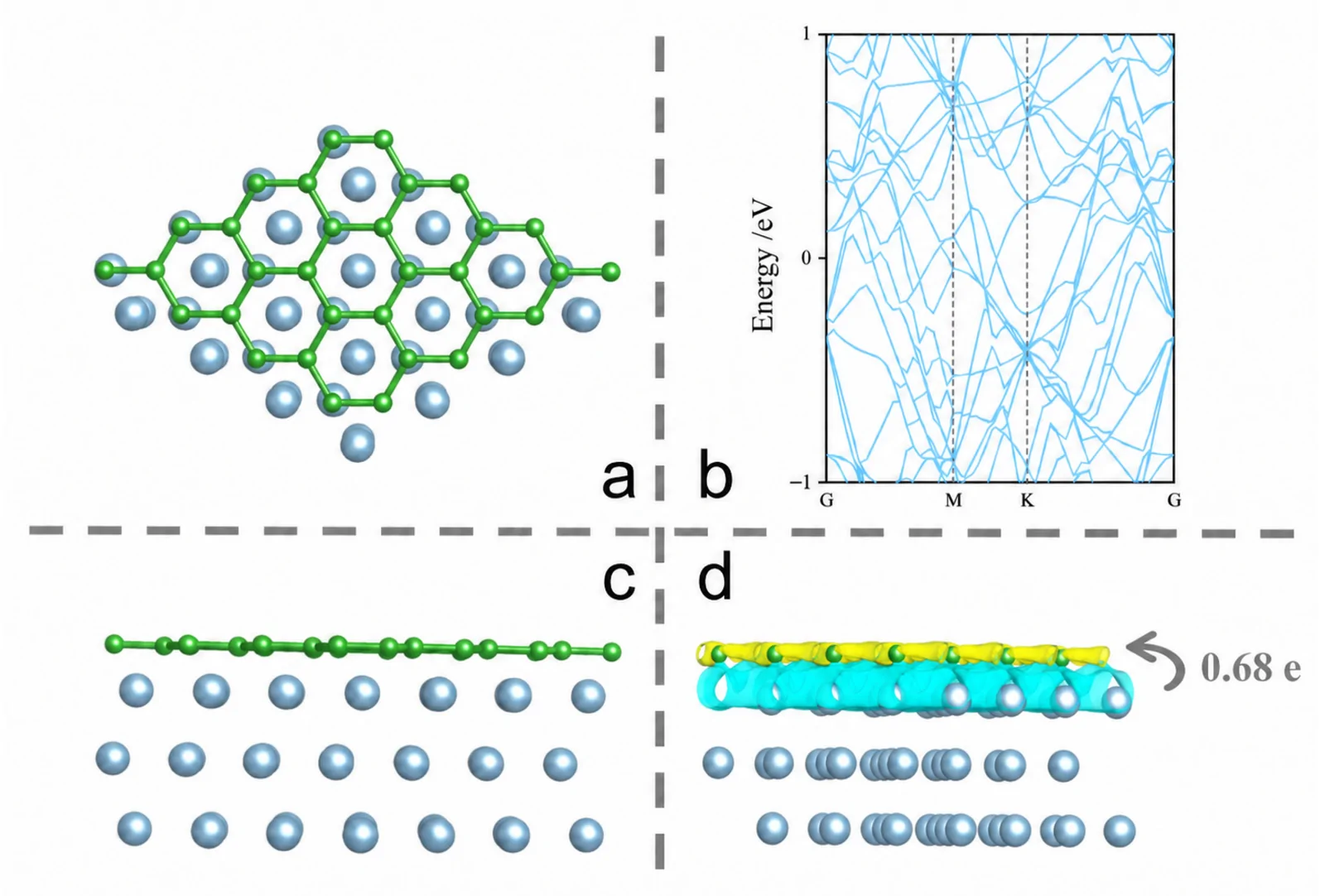
(**a**,**c**) Top and side views of the HB/Al(111) heterostructure, where green represents the B atoms and blue represents the Al atoms; (**b**) a band structure diagram of HB/Al (111) and (**d**) a differential charge diagram for HB/Al (111), with yellow indicating charge accumulation and blue indicating charge depletion.

As shown in Figure 1a,c, HB/Al(111) consists of four atomic layers: HB is the top layer, and the remaining three layers are stacked Al(111) surfaces. After geometric optimization, the B-B bond length is approximately 1.66 Å, and the vertical distance between B atoms and Al atoms is about 1.53 Å, which aligns with the data obtained from an experiment [38,39]. Figure 1b shows that multiple valence bands of HB/Al(111) cross the Fermi level, indicating its excellent conductivity. Meanwhile, the differential charge results (Figure 1d) indicate that each B atom can obtain approximately 0.68 electrons from the Al(111) substrate, suggesting a distinct chemical bond between HB and the Al(111) substrate that cannot be likened to ordinary van der Waals forces.

**Figure 2:**
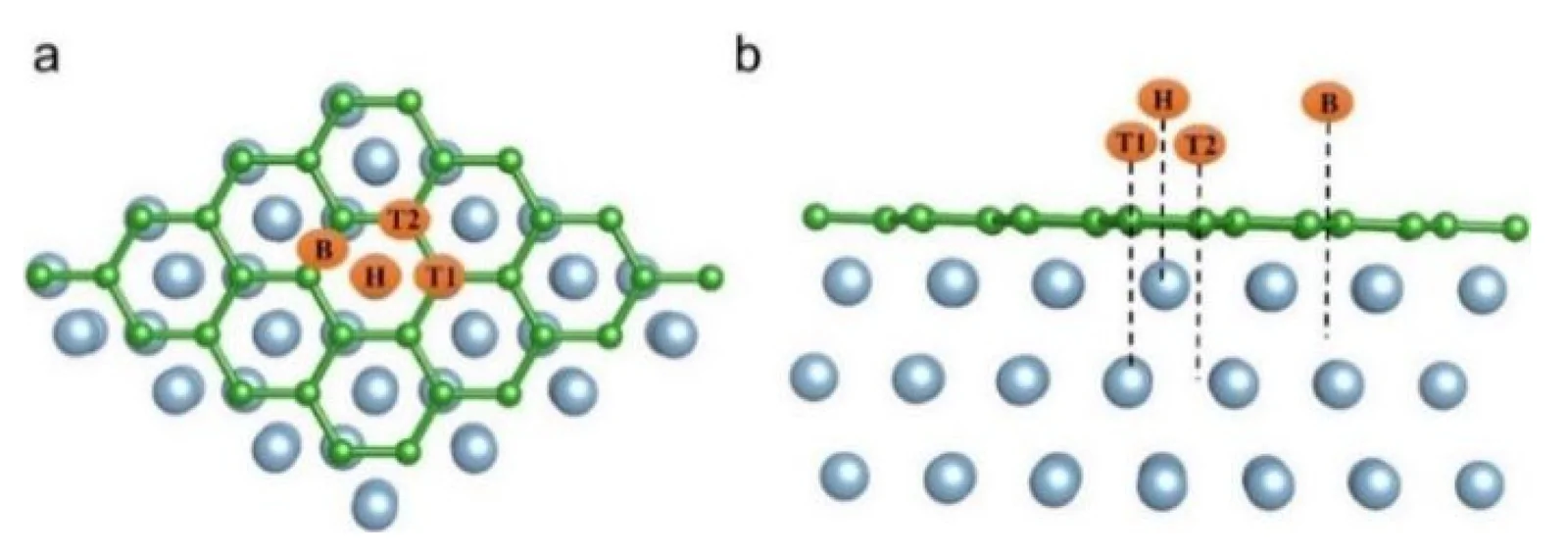
(**a**,**b**) Top and side views of four sites where metal atoms adsorb on the surface of HB/Al(111). Here, H, T1, T2, and B represent the Hollow, Top1, Top2, and Bridge sites, respectively.

Based on the stable HB/Al(111) structure, M atom doping was further evaluated. Previous studies have shown that there are four types of sites for a single metal atom adsorbing on the HB/Al(111) surface (Figure 2): the Hollow site, Top1 site, Top2 site and Bridge site, labelled H, T1, T2 and B, respectively. In this section, a total of eleven main-group metal atoms were chosen to build M-HB/Al(111) catalysts, including alkali metals (Li, Na, K, Rb), alkaline earth metals (Be, Mg, Ca, Sr), and p-block metals (Al, Ga, Ge).

As shown in Table S1, all metal atoms exhibit the lowest adsorption energy at the Hollow site, indicating their preference for adsorption at this site. Figure S1 depicts the charge of metal atoms after doping on HB/Al(111). From the diagram, it can be seen that all 11 metal atoms exhibit significant electron transfer to the substrate, demonstrating that metal atoms exist in the form of ions in the M-HB/Al(111) catalyst. Meanwhile, the alkaline earth metal atoms show the highest charge transfer amount, corresponding to the lowest adsorption energy. For alkali metals and p-block metals, there is no significant correlation between adsorption energy and atomic charge transfer. Interestingly, the transfer charge amounts are the same for all four alkali metal atoms, but there are significant differences in their adsorption energy values.

**Figure 3:**
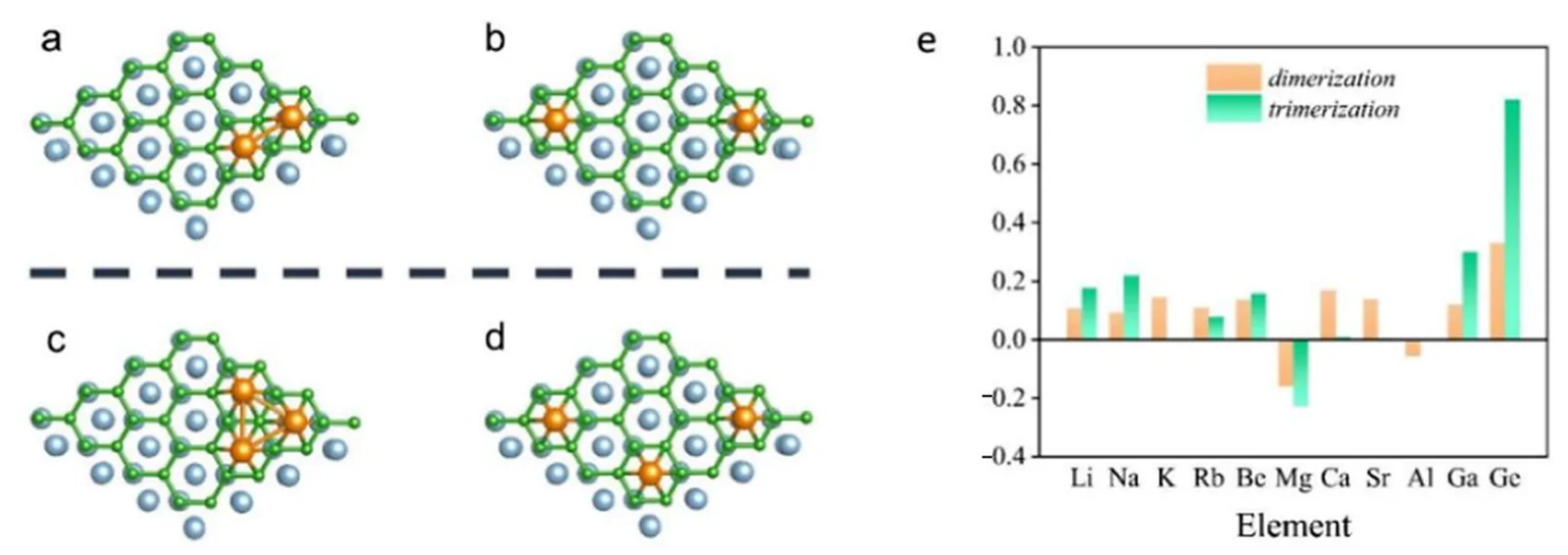
The typical configurations of two or three metal atoms adsorbed on the surface of HB/Al(111) during nucleation and separation: (**a**,**b**) depict two atoms, while (**c**,**d**) show three atoms. Here, blue represents Al atoms, green represents B atoms, and yellow represents Li atoms after color modification. (**e**) Differences in dimerization/trimerization energy for eleven different metals.

The stability of catalysts is an important indicator that must be considered in research. Previous studies have shown that although SACs can exhibit high catalytic activity, they are prone to aggregation and cluster formation, which can damage the overall structure of the catalysts [40,41]. Therefore, it is crucial to discover whether single atoms aggregate on the support. Firstly, the interaction of two metal atoms adsorbed on the substrate was investigated for two typical configurations. As shown in Figure 3a,b, one involved two atoms nucleating to form an M_2_ dimer, with an M-M distance of 3.14 to 6.29 Å, while the two metal atoms were far apart in the other configuration, at distance ranging from 9.95 to 19.72 Å. Here, the dimerization energy (ΔE_d_) was defined as E_dimer_ − E_separation_. E_dimer_ represents the total energy of the system when metal atoms are in a dimer state, while E_separation_ denotes the total energy of the system when metal atoms are in a separated state. Therefore, a positive ΔE_d_ indicates that metal atoms tend to be more dispersed on the HB/Al(111) surface. Conversely, a negative ΔE means that metal atoms prefer to form dimers on the HB/Al(111) surface. As shown in Figure 3e, except for the Mg- and Al-doped catalysts, which exhibit significant negative values, all of the doping systems have positive ΔE_d_ values. This demonstrates that the remaining nine metal atoms are capable of forming SACs on the HB/Al(111) surface. In addition, the trimerization energy among three atoms on the HB/Al(111) surface (Figure 3c,d) was calculated, and the results exhibit that Mg atoms trimerize rather than disperse on HB/Al(111). Therefore, nine metal single atoms (Li, Na, K, Rb, Be, Ca, Sr, Ga, and Ge) were selected to be adsorbed on the surface of HB/Al(111) to construct e-NitRR SACs.

**Figure 4:**
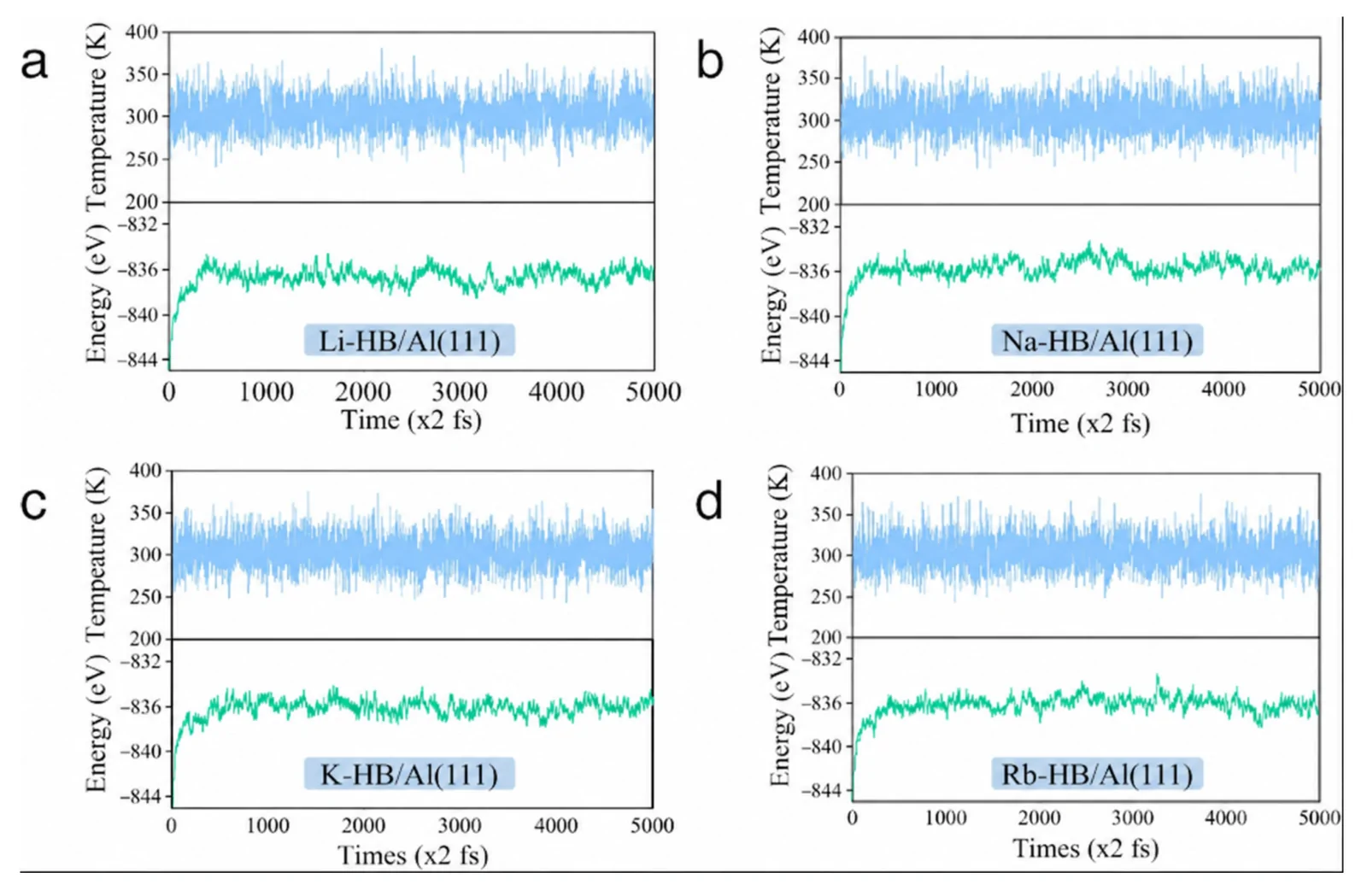
AIMD simulation results showing the temperature and total energy evolution of (**a**) Li-HB/Al(111), (**b**) Na-HB/Al(111), (**c**) K-HB/Al(111), and (**d**) Rb-HB/Al(111) at 300 K. The blue and green curves represent temperature and total energy, respectively.

In addition to their thermodynamic stability, the kinetic stability of catalysts also needs to be evaluated. Here, taking alkali metals as examples, AIMD simulations were conducted on four catalysts: Li-, Na-, K-, and Rb-HB/Al(111) (Figure 4a–d). The results show that after simulation at 300 K for 5 ps, the energy of the four catalysts converges while maintaining stable structures, indicating their kinetic stability. Therefore, the designed M-HB/Al (111) may serve as a stable e-NitRR catalyst.

### 3.2. Adsorption of NO_3_^−^ on M-HB/Al(111) Catalyst

**Figure 5:**
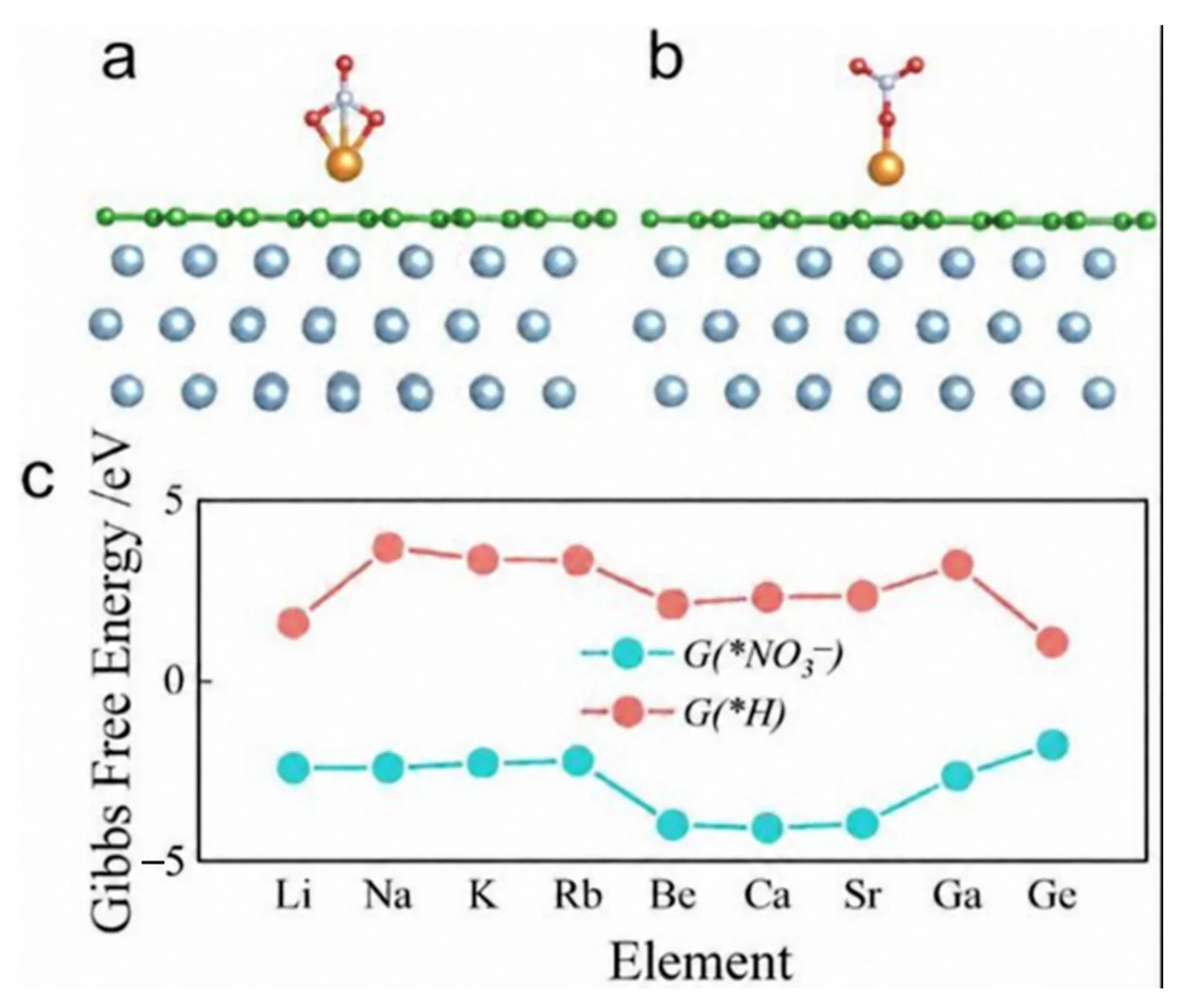
(**a**) The 2-O configuration of NO_3_^−^ adsorption and (**b**) the 1-O configuration of NO_3_^−^ adsorption. Here, blue represents Al atoms, green represents B atoms, yellow represents Li atoms, gray represents N atoms, and red represents O atoms. (**c**) Gibbs free-energy diagrams for the adsorption of NO_3_^−^ and H^+^ protons on 9 M-HB/Al (111) species. “*” represents the M-HB/Al(111) catalysts.

The adsorption of NO_3_^−^ in the initial step plays a critical role in the entire NO_3_^−^ reduction process. Generally, there are two adsorption configurations for NO_3_^−^ on a single atom: the 2-O configuration (the metal atom connects two O atoms) (Figure 5a) and the 1-O configuration (the metal atom connects one O atom) (Figure 5b). Calculations show that NO_3_^−^ exhibits a lower binding energy in the 2-O adsorption configuration on the M-HB/Al(111) catalysts. Figure 5c depicts the Gibbs free energy for NO_3_^−^ adsorption on the nine M-HB/Al (111) catalysts. For comparison, the Gibbs free energies for the adsorption of H protons were also calculated. The results show that the adsorption free energy of NO_3_^−^ on all M-HB/Al(111) catalysts is significantly negative, while ΔG(*H) is significantly positive. This exhibits that M-HB/Al(111) catalysts can not only effectively adsorb NO_3_^−^, laying a foundation for subsequent steps, but also exclude the possibility of the HER as a competitive reaction.

### 3.3. Mechanism of e-NitRR on M-HB/Al(111) Catalyst

Previous studies have shown that the binding strength between NO_3_^−^ and catalysts can serve as a scale for e-NitRR activity [42,43,44]. When ΔG_(*NO3-)_ is approximately −2 eV, the active sites of catalysts can demonstrate considerable catalytic activity. It can be seen from Figure 5c that the free energy of alkali metal atoms adsorbing NO_3_^−^ is around −2 eV, implying that they can exhibit high e-NitRR catalytic activity. Although the mechanism of the e-NitRR is very complex and there are many types of by-products (NO_2_, NO, N_2_O, etc.), according to the Pourbaix chart of nitrogen species, NH_3_/NH_4_^+^ has been confirmed as the thermodynamically most stable product under negative electrode potential [44]. Therefore, the study of the process of the e-NitRR reducing NO_3_^−^ to NH_3_ is of great significance.

**Figure 6:**
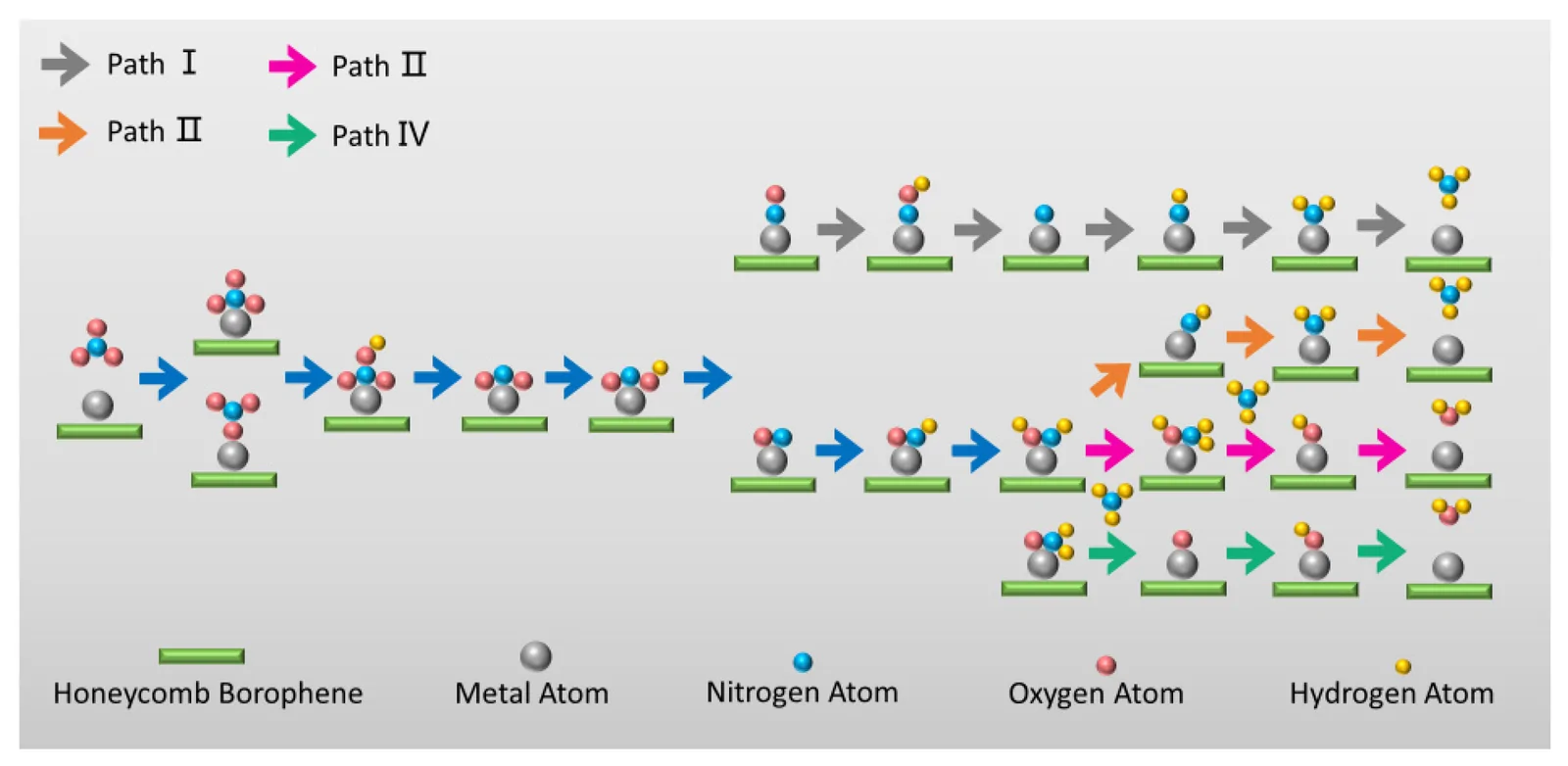
Schematic diagram of pathways for e-NitRRs via M-HB/Al(111).

The complete e-NitRR pathway is presented in Figure 6. Since the e-NitRR process contains a large number of intermediate configurations as well as hydrogenation sites, all four reaction pathways of these nine M-HB/Al(111) catalysts were taken into account in order to ensure that the optimal pathway could be determined. U_L_ is commonly used to evaluate the reaction activity of electrocatalytic reactions, and the e-NitRR limiting potential of seven M-HB/Al(111) catalysts is shown in Figure S2. From the graph, it can be observed that the U_L_ value demonstrates a consistent upward trend, indicating growth within the same main group. Following the first deoxygenation, the calculated NO_2_ adsorption energies on Ga-HB/Al(111) and Ge-HB/Al(111) are +1.89 and +1.32 eV, respectively (Figure S3). These positive values indicate energetically unfavorable NO_2_ adsorption relative to the adopted reference states. Therefore, the subsequent reduction pathways on these two catalysts were not investigated further. It is worth noting that the U_L_ of the Li-HB/Al(111) and Na-HB/Al(111) catalysts exhibited surprisingly low values, both around −0.1 V. This challenges the traditional research perspective that alkali metals cannot function as active sites in electrocatalytic reactions. Such low limiting potential is much smaller than those of transition metals that have been previously studied as active centers for reducing NO_3_^−^, including Ti/g-CN [45], Ru/g-C_3_N_4_ [46], V/g-C_3_N_4_ [47], Os/GDY [48], V/h-BP [49] and Fe/NG [50], for which the UL values are −0.39, −0.34, −0.25, −0.37, −0.22 and −0.38 V, respectively.

**Figure 7:**
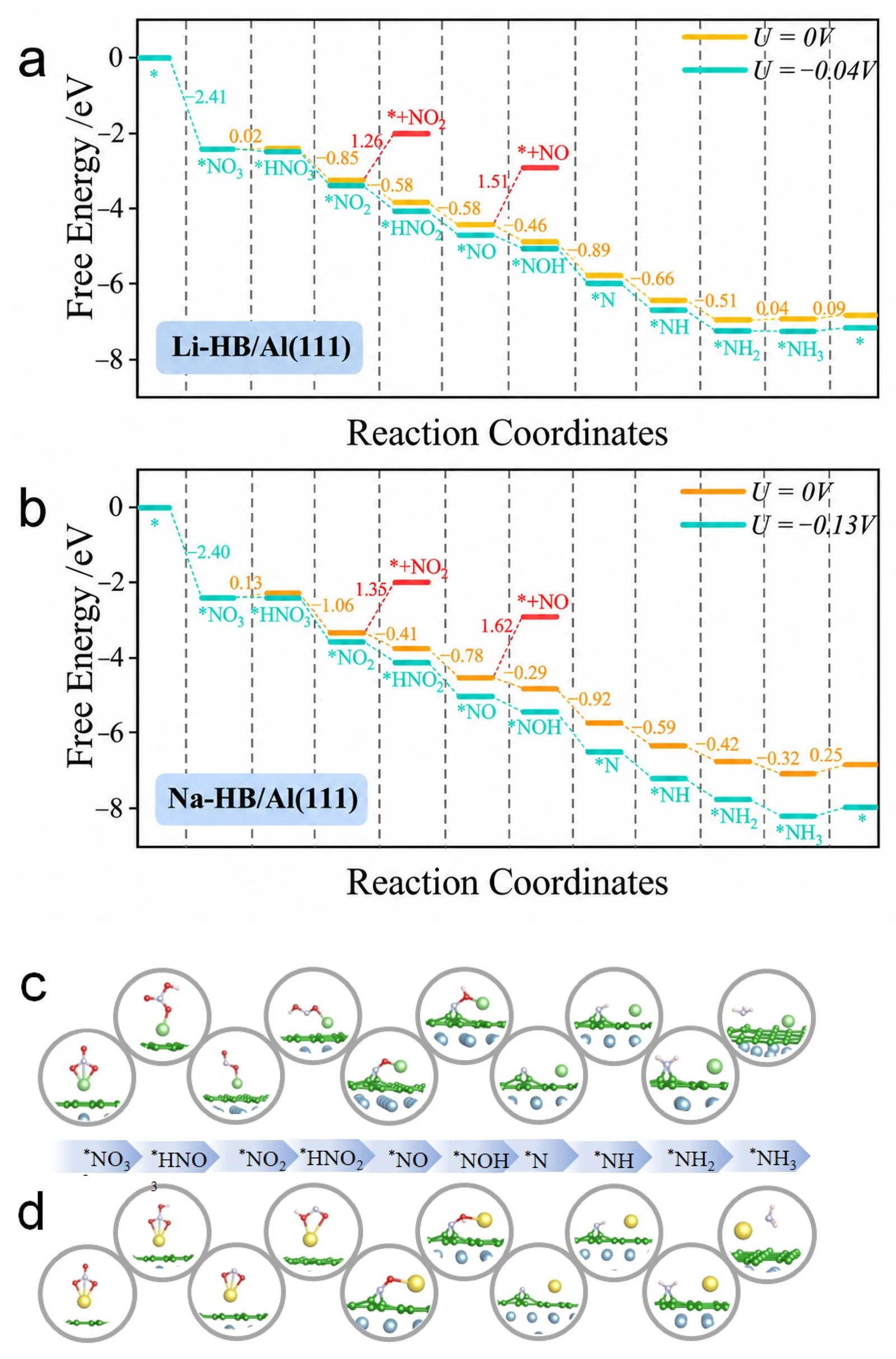
(**a**) Gibbs free-energy step diagrams of Li-HB/Al(111) and (**b**) Na-HB/Al(111) for e-NitRRs, where the dissociation of NO_2_ and NO has been taken into account; (**c**,**d**) the intermediate configurations involved in the reaction pathway, with (**c**) representing Li-HB/Al (111) and (**d**) representing Na-HB/Al(111). “*” represents the Na-HB/Al(111) or Li-HB/Al(111) catalyst.

To provide a deeper understanding of the mechanism of e-NitRRs, Figure 7 illustrates the reaction pathways and corresponding Gibbs free-energy profiles on Li-HB/Al (111) and Na-HB/Al(111) catalysts. In Figure 7a,b, the red lines represent the desorption pathways of NO_2_ and NO from the catalyst surfaces. The positive desorption free-energy changes indicate that the desorption of these species is thermodynamically unfavorable under the adopted reference conditions, providing a thermodynamic basis for evaluating their possible release as by-products. For the Li-HB/Al(111) catalyst, when NO_3_^−^ is adsorbed on the central metal atom, the free energy of the system decreases by 2.41 eV (Figure 7a). Subsequently, the first hydrogenation step occurs on the far-end O atom of NO_3_^−^, with a change in adsorption configuration (Figure 7c) and a slight increase in energy of only 0.02 eV. After the second hydrogenation step, the first water molecule is removed, leading to a decrease of 0.85 eV in free energy. NO_2_ is adsorbed on the catalyst in a V-shape via Li-O bonding. In the third hydrogenation step, which is similar to the first step, H^+^ attacks the remote O atom, resulting in M-shaped adsorption of the entire adsorbate and a decrease in free energy of 0.58 eV. When the fourth hydrogenation step occurs, the second water molecule is removed from the system, and the adsorption configuration undergoes a significant change. The N atom interacts with B atoms on the catalyst surface, while the O atom remains bonded to Li^+^. The system energy decreases by 0.58 eV. H^+^ attacks the N or O atom in the fifth hydrogenation step, leading to different paths. The energy to form the ^*^NOH adsorption configuration is favorable, with a 0.46 eV free-energy decrease. The following sixth, seventh, and eighth hydrogenation steps exhibit a decreasing trend in energy, with values of −0.89, −0.66, and −0.51 eV, respectively. In the final hydrogenation step (*NH_2_ → *NH_3_), the free energy slightly increases by 0.04 eV, making it the potential-determining step (PDS) for the entire reaction. The detailed hydrogenation process starting from the *NOH configuration is *NOH → *N + H_2_O (l) → *NH → *NH_2_ → *NH_3_ → *+NH_3_ (g).

Figure S4 shows the optimized configurations of the reaction intermediates in the presence of explicit water. The reaction free-energy profiles of Li-HB/Al(111) with and without explicit water at pH = 0 and U = 0 V versus SHE are shown in Figure S5. The free-energy change for *NO_3_ → *HNO_3_ decreases from 0.02 eV to −0.04 eV, whereas that for *NH_2_ → *NH_3_ increases from 0.04 eV to 0.06 eV. Among the examined electrochemical steps, the PDS remains the final hydrogenation step, giving a CHE limiting potential of −0.06 V versus SHE. The comparison indicates that solvent effects vary among intermediates rather than producing a uniform stabilization of the entire pathway. These results indicate that solvation affects different reaction states to varying degrees but has only a limited overall effect on the reaction thermodynamics.

Figure S7 compares the CHE-derived free-energy profiles at pH = 0 and 7 for Li-HB/Al(111), evaluated at U = 0 V versus SHE and 298.15 K. Increasing the pH raises the free-energy changes of proton-consuming steps. The free-energy changes for *NO_3_ → *HNO_3_ and *NH_2_ → *NH_3_ increase from 0.02 eV and 0.04 eV to approximately 0.43 and 0.45 eV, respectively. Within the retained pathway and reference-state conventions, *NH_2_ → *NH_3_ remains the PDS, with a maximum uphill reaction free-energy change of approximately 0.45 eV at pH = 7. These results describe the pH dependence at a fixed SHE potential.

Overall, the mechanism of e-NitRRs catalyzed by Na-HB/Al(111) is similar to that on Li-HB/Al(111), including reaction pathways, energy changes, and intermediate configurations. The difference lies in the fact that the free energy in the second hydrogenation step, which is the PDS of the whole reaction, increases by 0.13 eV (Figure 7b). Only this hydrogenation step in the entire path shows an upward trend. According to what we mentioned in the previous section, five of the nine M-HB/Al(111) catalysts exhibit e-NitRR limit potentials below the standard values. The Gibbs free-energy step diagrams of the remaining three catalysts are shown in Figure S6 and are not elaborated here due to their similar reaction mechanism.

### 3.4. e-NitRR Selectivity

Selectivity has always been an essential criterion for evaluating electrochemical reduction reactions. In addition to the HER mentioned above, the process of electrochemical reduction of NO_3_^−^ may also produce various by-products, including NO_2_, NO, N_2_O, and N_2_. From Figure 7a,b, it can be observed that for both the Li-HB/Al(111) and Na-HB/Al(111) catalysts, the adsorption energies of NO_2_ and NO are approximately 1.5 eV, significantly higher than the energy needed for the corresponding e-NitRR PDS. Therefore, the possibility of NO_2_ and NO being produced as by-products is ruled out. The NO_2_ and NO desorption free-energy changes increase from 1.26 and 1.51 eV to 1.44 and 1.61 eV, respectively. The calculated NH_3_ desorption free-energy changes are +0.09 and +0.25 eV for Li-HB/Al(111) and Na-HB/Al(111), respectively, indicating that NH_3_ release is thermodynamically uphill under the adopted reference conditions. These values describe the thermodynamic cost of desorption rather than desorption activation barriers. On the other hand, N_2_O_2_ with a cis-O=N-N=O structure is a precursor for the formation of N_2_O and N_2_, connected by two NO molecules [51]. However, due to the isolated single atomic site in the studied catalysts, it is impossible to simultaneously combine two NO_3_^−^ molecules. Therefore, the Langmuir–Hinshelwood mechanism for the formation of NO dimers has been excluded [52]. Meanwhile, as NO tends to undergo hydrogenation rather than dissociation, another Eley–Rideal mechanism for forming NO dimers is also not considered [53]. In summary, we can conclude that none of the four byproducts (NO_2_, NO, N_2_O, and N_2_) have the possibility to be generated during e-NitRRs on Li-HB/Al(111) and Na-HB/Al(111) catalysts.

### 3.5. Activation of NO_3_^−^ on M-HB/Al(111)

Based on the previous analysis, it can be inferred that the binding strength between NO_3_^−^ and the catalytic active center can predict the activity trend of the e-NitRR. Figure S2 illustrates that different M-HB/Al(111) catalysts exhibit significant variations in their limiting potentials. Therefore, it can be speculated that this difference is linked to the activation of NO_3_^−^. To verify this speculation, Li-HB/Al(111) with the lowest limiting potential (−0.04 V) and Sr-HB/Al(111) with the highest limiting potential (−1.07 V) were chosen for comparison.

**Figure 8:**
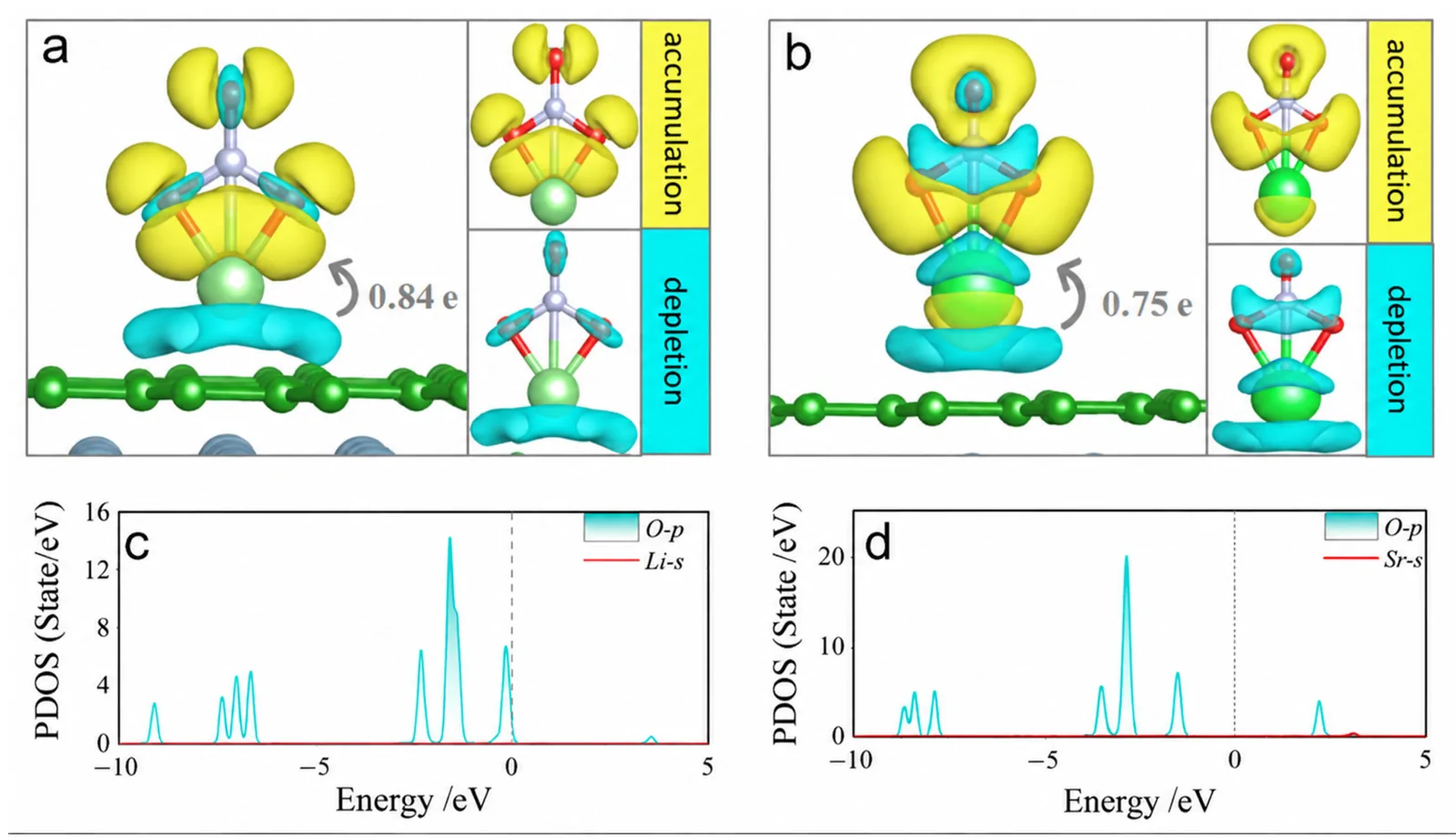
Differential charge diagrams of (**a**) Li-HB/Al(111) and (**b**) Sr-HB/Al (111) after NO_3_^−^ adsorption. The isosurface value is 0.0015 e/Å^3^. PDOS diagrams after NO_3_^−^ adsorption on (**c**) Li-HB/Al(111) and (**d**) Sr-HB/Al(111).

The differential charge diagrams of the two catalysts with NO_3_^−^ adsorption are presented in Figure 8a,b. The metal atoms in the M-HB/Al(111) catalyst transfer a substantial amount of electrons to NO_3_^−^, leading to their adsorption on the HB/Al(111) surface in an ionic form (Li carries 0.88 positive charges, Sr carries 1.82 positive charges). This implies that the active center metal ion bonds with NO_3_^−^ through ionic interaction. Calculations show that electrons acquired by NO_3_^−^ adsorbed on the two catalysts both amount to 0.85 e, with the HB/Al(111) substrate acting as an electron donator. The difference originates from the fact that on Li-HB/Al(111), almost all the electrons obtained by NO_3_^−^ are provided by the HB/Al(111) substrate (0.84 e) and Li^+^ acts as a bridge for transporting electrons, but for NO_3_^−^ on Sr-HB/Al(111), 0.75 electrons are transferred from HB/Al(111) and the remaining 0.1 electrons are donated by Sr. This observation suggests the existence of a slight covalent interaction between Sr and NO_3_^−^, enhancing the adsorption of Sr-HB/Al(111) for NO_3_^−^. This is the reason why the adsorption energies of the two systems are very different even though the number of electrons obtained by NO_3_^−^ is comparable (ΔG(*NO_3_^−^) values are −2.41 eV and −3.95 eV for Li-HB/Al(111) and Sr-HB/Al(111) respectively). The strong adsorption leads to a higher reaction Gibbs free-energy change for subsequent hydrogenation steps of NO_3_^−^. Furthermore, analysis of the differential charge density map reveals that simultaneous donation–feedback of electrons occurs on NO_3_^−^. For instance, in Figure 8a, the electron density around the O atom increases as electrons acquired by NO_3_^−^ occupy the antibonding orbitals of the O atom, whereas NO_3_^−^ loses electrons from its bonding orbitals. This mechanism resembles the “acceptor–donator” mechanism, activating NO_3_^−^ on the metal ion catalytic center.

Further, the activation mechanism of NO_3_^−^ can also be analyzed in the density of states (DOS). As depicted in Figure 8c,d, p orbitals of the O atom in NO_3_^−^ linked to the active center exhibit a substantial number of electron states below the Fermi level, whereas the s orbitals of Li and Sr do not. Observation suggests that M-HB/Al(111) stabilizes NO_3_^−^ mainly through ionic bonding, aligning with the above analysis. Furthermore, for NO_3_^−^ adsorption on Li-HB/Al(111), the HOMO energy of the O atom is notably higher compared to that on Sr-HB/Al(111), indicating a more pronounced activation extent.

**Figure 9:**
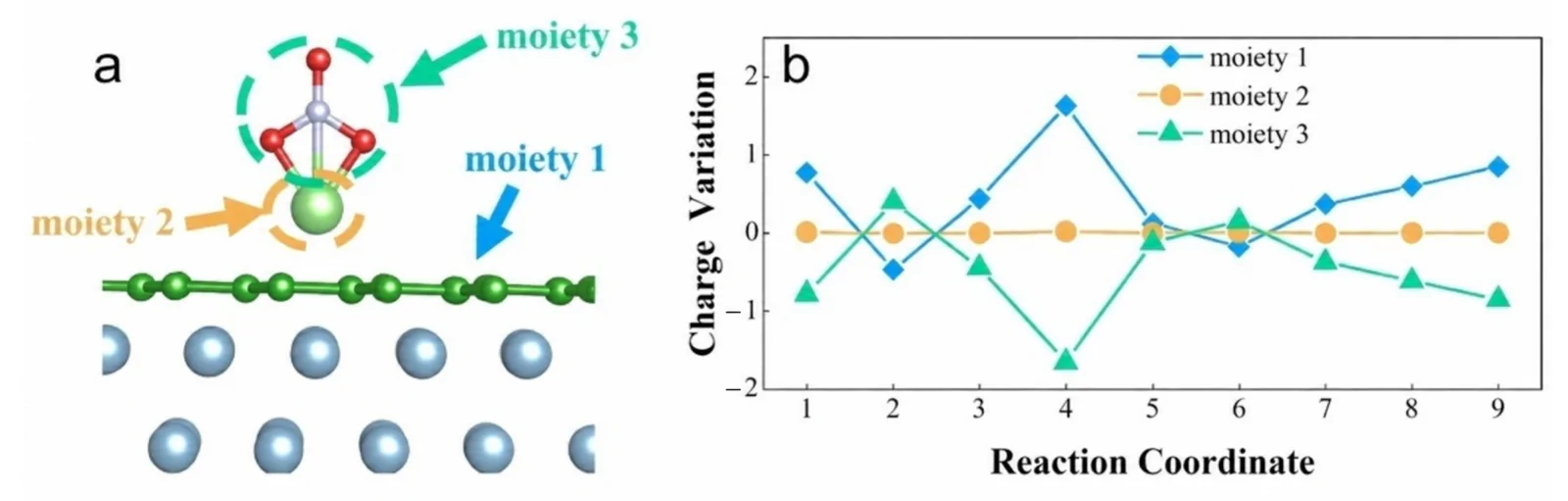
(**a**) The adsorption structure of NO_3_^−^ on Li-HB/Al(111); (**b**) variation in the atomic charge compared to the previous step along the e-NitRR pathway catalyzed by Li-HB/Al(111). Moieties 1, 2 and 3 represent HB/Al(111), Li^+^, and NO_3_^−^, respectively.

Finally, to further explain the high catalytic activity demonstrated by the M-HB/Al(111) catalysts in e-NitRRs, the charge transfer process during the whole e-NitRR pathway on Li-HB/Al(111) was analyzed. The adsorption system of NO_3_^−^ on Li-HB/Al(111) is divided into three parts (Figure 9a): the HB/Al(111) substrate as moiety 1, Li^+^ as moiety 2, and NO_3_^−^ as moiety 3 [54]. The charge changes along the entire pathway are illustrated in Figure 9b, where reaction coordinates 1–9 represent the hydrogenation step number of the e-NitRR. It can be observed that there is no significant charge transfer for the lithium ion during the reaction process, confirming the mechanism we discussed earlier: the active center binds with NO_3_^−^ through ionic bonds and serves as a bridge for charge transfer from the substrate to NO_3_^−^. Since Li already carries a 0.78 positive charge before NO_3_^−^ adsorption, it can be confirmed that the lithium ion maintains a positive charge throughout the entire e-NitRR process, which plays a significant role in stabilizing intermediates and preventing H poisoning.

### 3.6. Descriptor of M-HB/Al(111) Catalytic Activity in e-NitRR

Catalytic performance descriptors can help establish the relationship between the structure of a catalyst and its catalytic performance, reducing a complex reaction process to a few key physicochemical parameters [55,56,57]. Based on these descriptors, researchers can make a preliminary judgment on the possible activity trend of the catalyst during the whole reaction process without having to consider the details of each microscopic reaction step. Descriptors can also be used to predict the performance of catalysts that have not yet been experimentally validated, greatly improving the efficiency of catalyst screening and reducing experimental cost.

**Figure 10:**
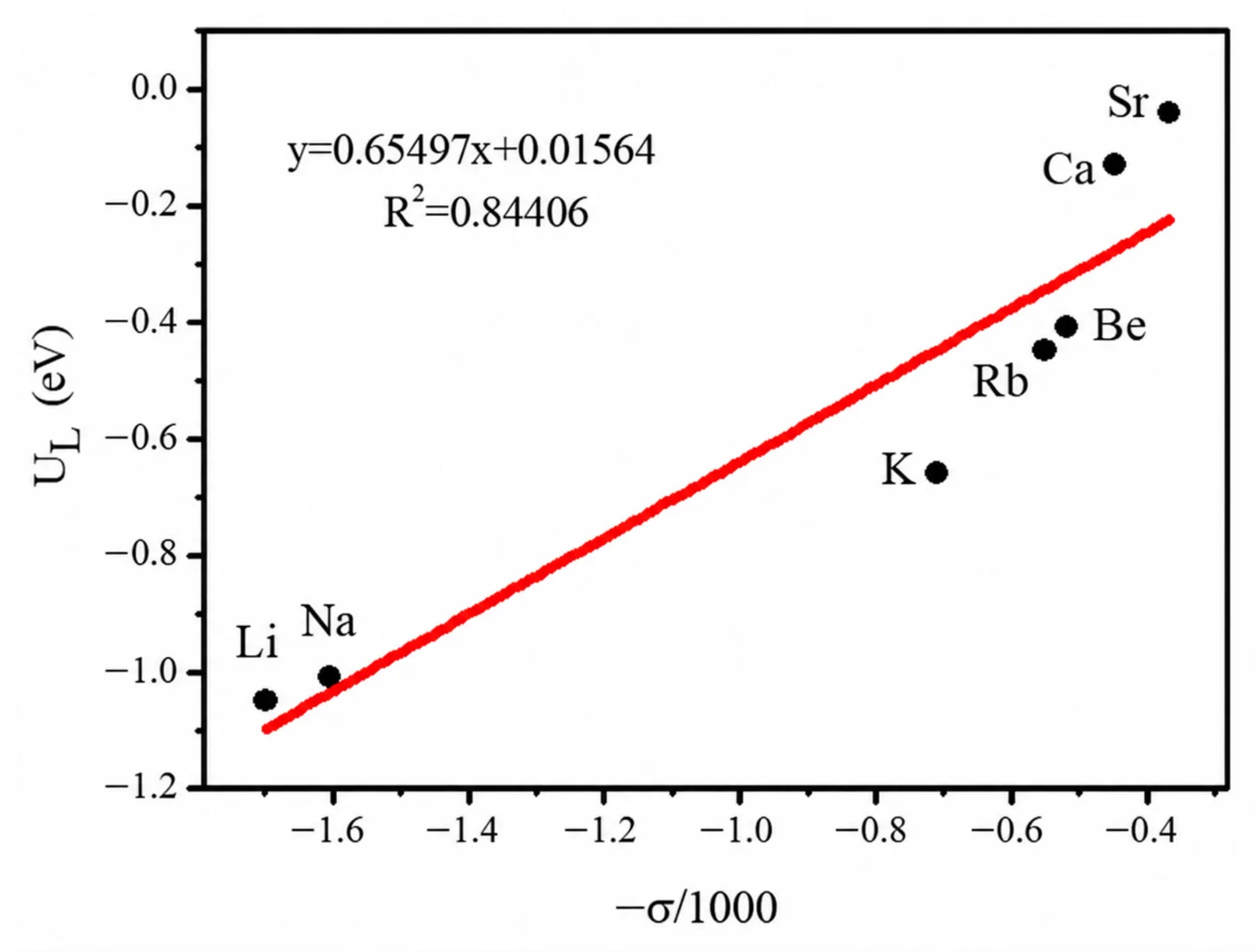
Curve of relationship between e-NitRR limiting potential U_L_ and −σ/1000.

Generally, the adsorption energy of a reactant on a catalyst can considerably affect the subsequent reaction. Besides this, the structural and electronic properties of doping atoms in SACs also play an important role in reaction performance. Therefore, the descriptor $\sigma =G{(*\mathrm{NO}}_{3}^{-})\cdot R\cdot Z$ was built up to describe the reaction catalytic activity, where $G{(*\mathrm{NO}}_{3}^{-})$ is the adsorption free energy of reactant NO_3_^−^ on the catalyst, while R and Z represent the atomic radius and valence electron number of the doping atoms. For clarity, a graph of the correlation between the reaction limiting potential and descriptor was plotted with -σ/1000 as the horizontal axis. Figure 10, for the studied reaction systems, exhibits a strong linear correlation between limiting potential and the descriptor, with R^2^ = 0.844, suggesting that σ is a reliable descriptor for the reaction performance. And importantly, the three related parameters—the NO_3_^−^ adsorption free energy, along with the atomic radius and valence electron number of the doping atom—contained in the descriptor are easy to obtain, which provides a convenient way to screen and rationally design catalysts for e-NitRRs.

### 3.7. Competition with the Hydrogen Evolution Reaction

**Figure 11:**
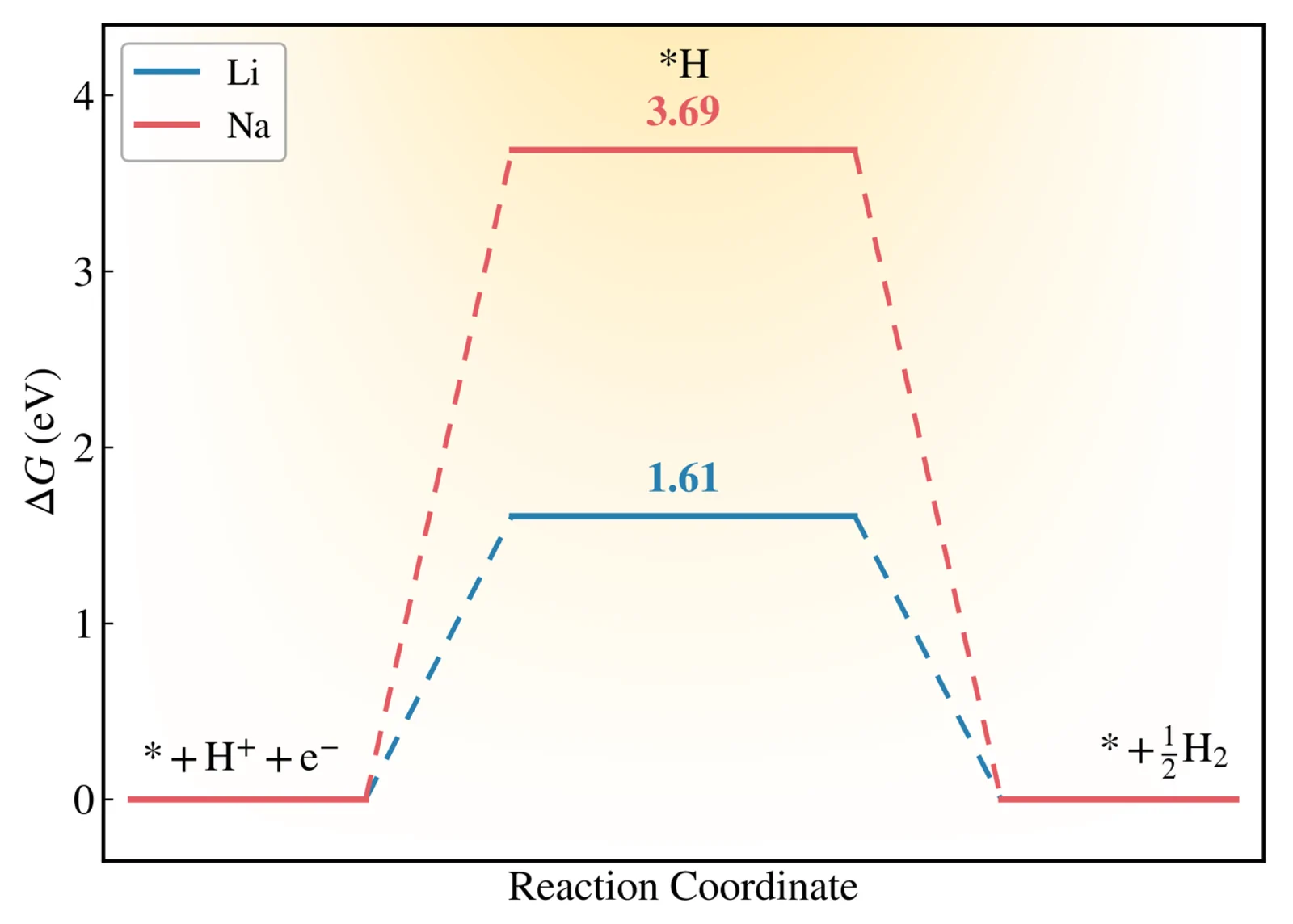
Hydrogen adsorption free energies at the Li and Na sites. “*” represents the Na-HB/Al(111) or Li-HB/Al(111) catalyst.

In addition to nitrate reduction activity, competition from the hydrogen evolution reaction (HER) is an important consideration in evaluating electrocatalysts [58]. We therefore calculated the hydrogen adsorption free energies (ΔG_*H_) on Li-HB/Al(111) and Na-HB/Al(111). As shown in Figure 11, their ΔG_*H_ values are +1.61 and +3.69 eV, respectively, substantially above the thermoneutral value of 0 eV commonly used as an HER activity descriptor. These positive values indicate that *H formation is thermodynamically unfavorable at the investigated sites under the reference conditions. Together with the small maximum uphill free-energy changes for nitrate reduction, these results suggest a thermodynamic preference for nitrate reduction over hydrogen adsorption.

## 4. Conclusions

In this work, a series of M-HB/Al(111) (M = Li, Na, K, Rb, Be, Mg, Ca, Sr, Al, Ga, and Ge) electrocatalysts composed of M atoms and an HB/Al(111) heterostructure were theoretically constructed. The thermodynamic and kinetic stability of these catalysts were verified by DFT binding energy computations and AIMD simulations. The aggregation tests identified nine stable M-HB/Al(111) SAC candidates. Through calculations of the reaction mechanism and free energy, it was discovered that Li-HB/Al(111) and Na-HB/Al(111) exhibit very high catalytic activity, with limiting potentials of −0.04 V and −0.13 V respectively, which breaks through the traditional perspective that alkali metals cannot function as catalytic active centers. Research on the activation mechanism suggests that the function of M-HB/Al(111) in reducing NO_3_^−^ lies in the active center’s ability to fix NO_3_^−^ through ionic bonding interaction, while M ions serve as a bridge transporting electrons from the substrate to the adsorbate. A new descriptor was confirmed to form a linear scaling relationship with the limiting potential of the catalytic e-NitRR. This work may open a new avenue for screening and designing novel and efficient main-group metal SACs.

## Data Availability

Data will be made available on request.

## Supplementary Materials

The following supporting information can be downloaded at https://www.mdpi.com/article/10.3390/molecules31213983/s1, Figure S1: Charge transfer histogram of hollow sites adsorbed by LM atoms on HB/Al (111) surface; Figure S2: The value of the limiting potential of e-NitRR corresponding to seven LM-HB/Al(111) catalysts; Figure S3: Structures before and after optimization starting from (a) N-bound and (b) monodentate O-bound configurations; Figure S4: Optimized stable configurations of the reaction intermediates in the presence of explicit solvent molecules; Figure S5: Reaction Gibbs free-energy profiles for nitrate reduction on Li-HB/Al(111) with and without explicit water at pH = 0 and U = 0 V versus SHE. Branches show NO_2_ and NO desorption. Values beside the dashed connectors are stepwise reaction free-energy changes in eV, not activation barriers; Figure S6: Gibbs free energy step diagrams of(a) K-HB/Al (111), (b) Rb HB/Al (111), and (c) Be HB/Al (111) for e-NitRR; Figure S7: Comparison of the CHE-derived Gibbs free-energy profiles for nitrate reduction on Li-HB/Al(111) at pH = 0 and 7, calculated at 298.15 K and U = 0 V versus SHE. Table S1: The adsorption energy of eleven light metal atoms at four sites on the surface of HB/Al (111). Among them, “/” represents that the site does not exist.
