# Supplementary material for: Main-Group Metal Atoms Anchored on Borophene/Al(111) as an Efficient Catalyst for Electrocatalytic Nitrate Pollutant Reduction Reactions: A First-Principle Perspective
Source: Molecules. 2026 Nov 4;31(21):3983. doi: 10.3390/molecules31213983 (PMC13765473; doi:10.3390/molecules31213983)
Supplement: Supplementary file 1 [file molecules-31-03983-s001.zip › molecules-4594847-supplementary.pdf]

Supporting Information

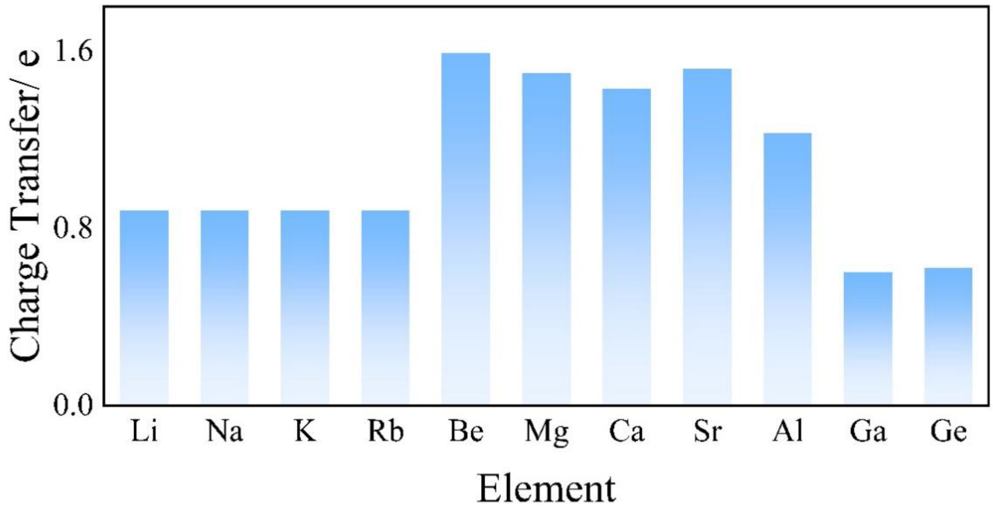

Figure S1. Charge transfer histogram of hollow sites adsorbed by LM atoms on HB/Al (111) surface

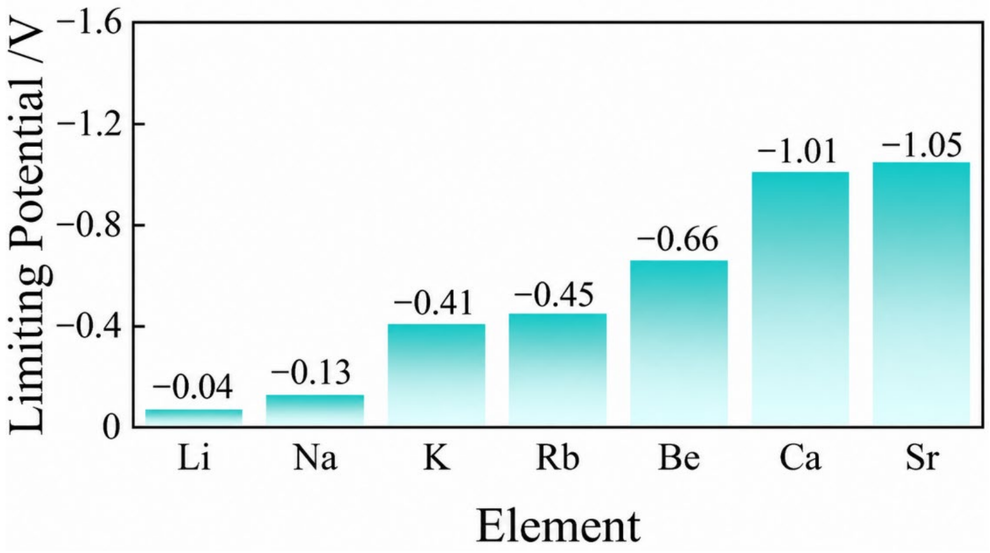

Figure S2. The value of the limiting potential of e-NitRR corresponding to seven LM-HB/Al(111) catalysts

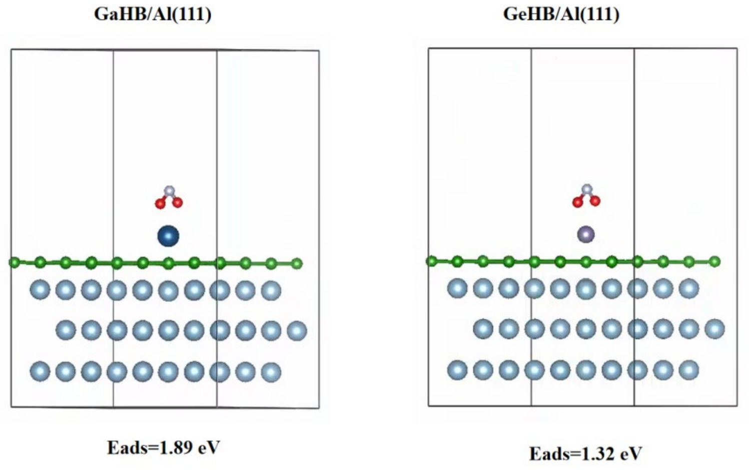

Figure S3. Structures before and after optimization starting from (a) N-bound and (b) monodentate O-bound configurations

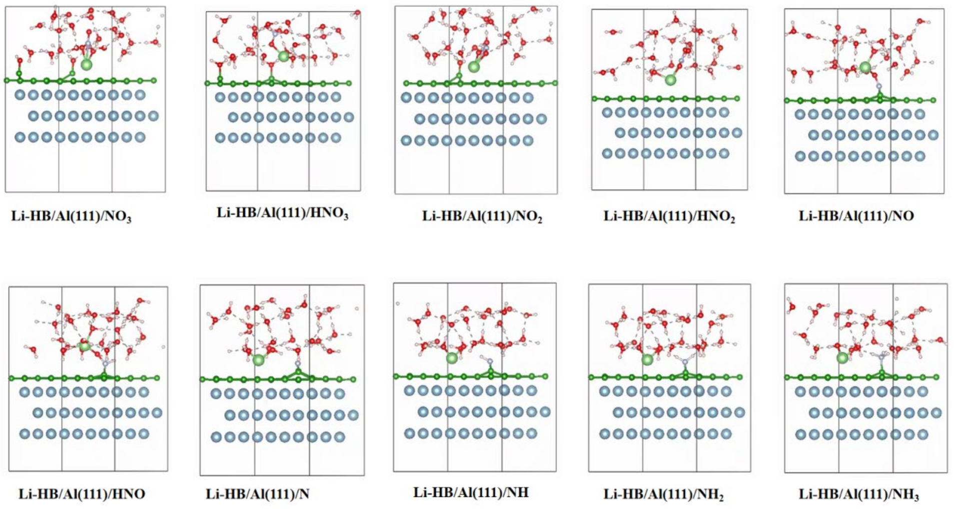

Figure S4. Optimized stable configurations of the reaction intermediates in the presence of explicit solvent molecules

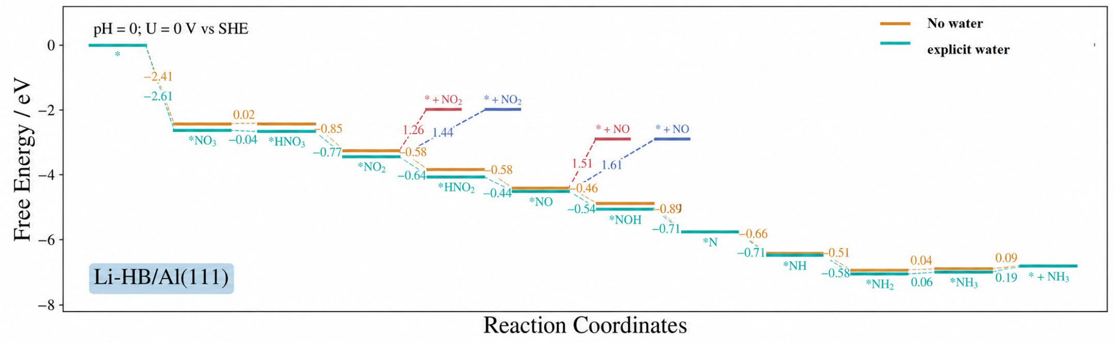

Figure S5. Reaction Gibbs free-energy profiles for nitrate reduction on Li-HB/Al(111) with and without explicit water at pH = 0 and U = 0 V versus SHE. Branches show NO_2 and NO desorption. Values beside the dashed connectors are stepwise reaction free-energy changes in eV, not activation barriers

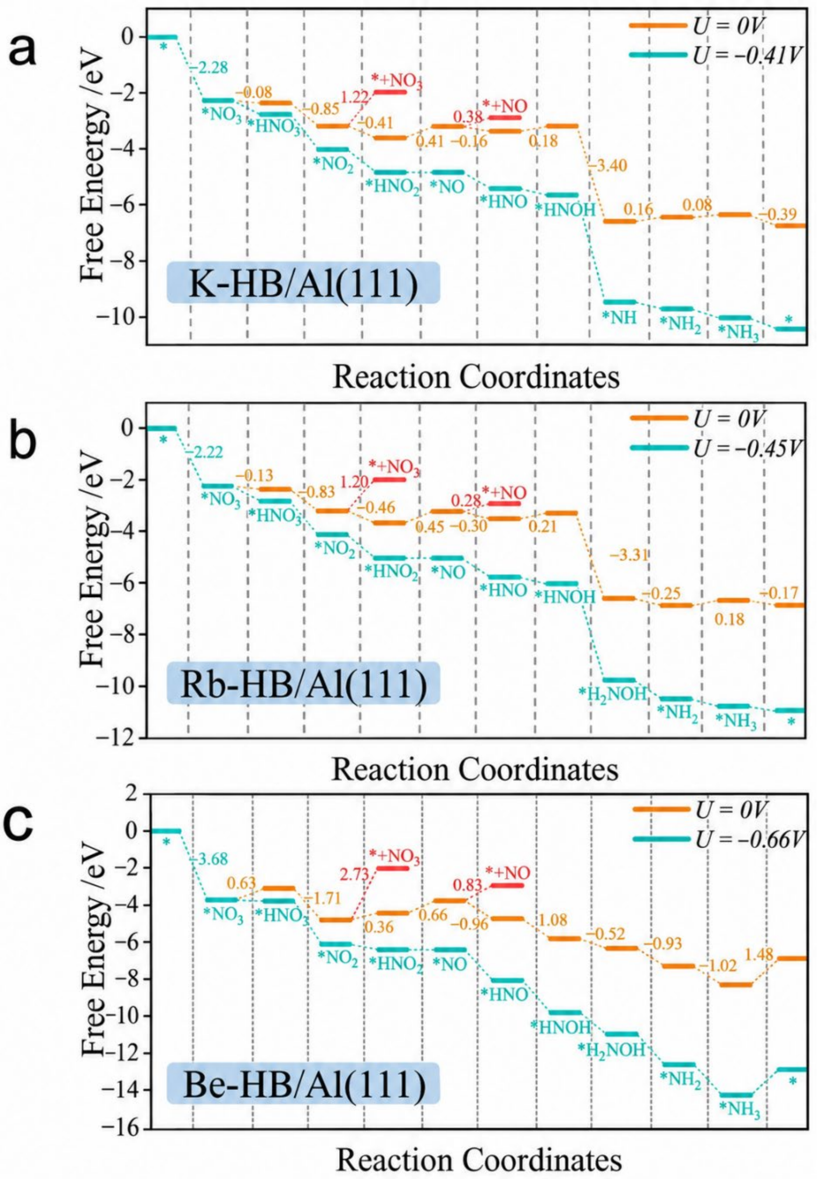

Figure S6. Gibbs free energy step diagrams of(a) K-HB/Al (111), (b) Rb HB/Al (111), and (c) Be HB/Al (111) for e-NitRR

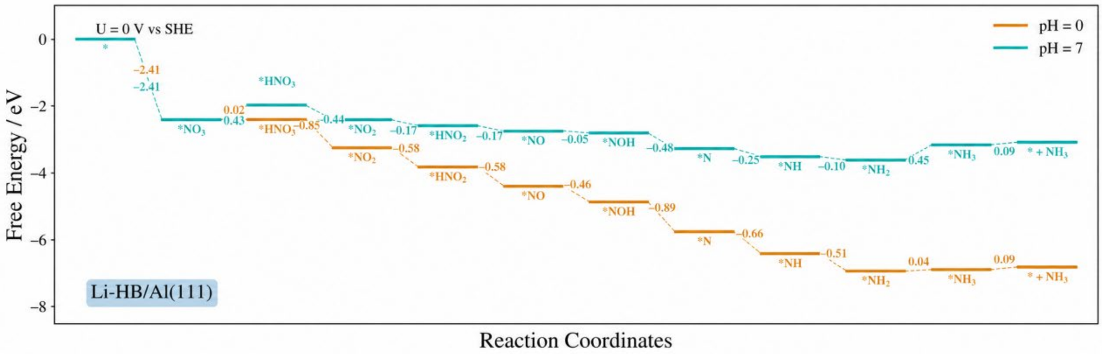

Figure S7. Comparison of the CHE-derived Gibbs free-energy profiles for nitrate reduction on Li-HB/Al(111) at pH = 0 and 7, calculated at 298.15 K and U = 0 V versus SHE

Table S1. The adsorption energy of eleven light metal atoms at four sites on the surface of HB/Al (111). Among them, "/" represents that the site does not exist

**Light Metals**	**Hollow (*eV*)**	**Top1 (*eV*)**	**Top2 (*eV*)**	**Bridge (*eV*)**
**Li**	−3.59	−3.04	−3.04	−3.58
**Na**	−2.72	−2.48	−2.48	−2.72
**K**	−2.97	−2.82	−2.83	/
**Rb**	−2.89	−2.78	−2.78	−2.79
**Be**	−4.43	/	/	−4.47
**Mg**	−2.06	/	6.09	−2.06
**Ca**	−3.99	/	/	/
**Sr**	−3.87	/	/	/
**Al**	−3.97	/	/	−3.96
**Ga**	−3.22	−2.83	−2.79	−2.83
**Ge**	−4.85	−3.48	−3.58	−4.27
